# Nanoscale Three-Dimensional Charge Density and Electric Field Mapping by Electron Holographic Tomography

**DOI:** 10.1021/acs.nanolett.2c03879

**Published:** 2023-01-23

**Authors:** Fengshan Zheng, Vadim Migunov, Jan Caron, Hongchu Du, Giulio Pozzi, Rafal E. Dunin-Borkowski

**Affiliations:** † Ernst Ruska-Centre for Microscopy and Spectroscopy with Electrons and Peter Grünberg Institute, Forschungszentrum Jülich, 52425 Jülich, Germany; ‡ Spin-X Institute, Electron Microscopy Center, School of Physics and Optoelectronics, State Key Laboratory of Luminescent Materials and Devices, Guangdong-Hong Kong-Macao Joint Laboratory of Optoelectronic and Magnetic Functional Materials, South China University of Technology, Guangzhou 511442, China; § Central Facility for Electron Microscopy (GFE), RWTH Aachen University, Ahornstrasse 55, 52074 Aachen, Germany; ∥ Department FIM, University of Modena and Reggio Emilia, via G. Campi 213/a, 41125 Modena, Italy

**Keywords:** charge density, electric field, three-dimensional mapping, electron holography, tomography

## Abstract

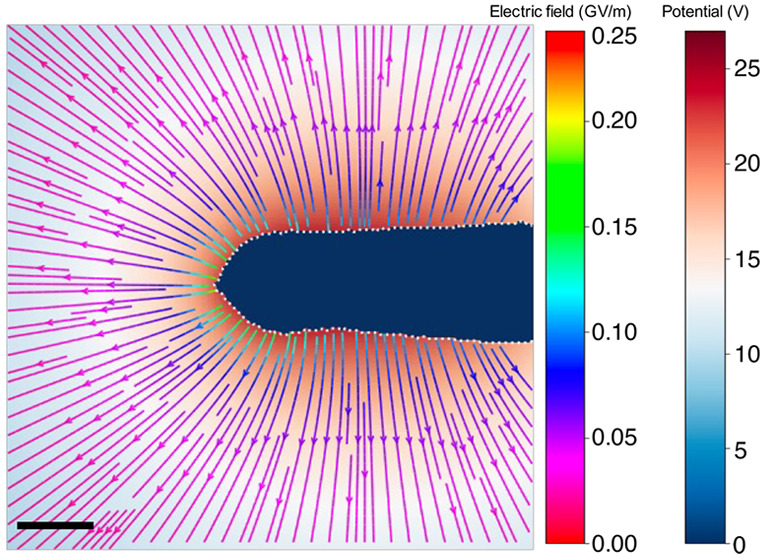

The operation of nanoscale electronic devices is related intimately to the three-dimensional (3D) charge density distributions within them. Here, we demonstrate the quantitative 3D mapping of the charge density and long-range electric field associated with an electrically biased carbon fiber nanotip with a spatial resolution of approximately 5 nm using electron holographic tomography in the transmission electron microscope combined with model-based iterative reconstruction. The approach presented here can be applied to a wide range of other nanoscale materials and devices.

Nanoscale electronic devices such as transistors and light-emitting diodes are widely used in high technology applications. In order to improve the working performance of such devices, it is important to understand the relationship between their local electrical properties and the presence of defects, dopants, interfaces, and surfaces.^1^ For example, the emission performance of electron sources in electron microscopes (in particular, field emitters) is determined by geometrical factors, including local shape, curvature, and crystallographic orientation.^2−4^ In atom probe tomography,^5^ the shape, chemistry, and defect distribution of a needle-shaped sample determine the electric field around it, influencing the trajectories of field-evaporated ions and affecting the fidelity of reconstructed atom positions.^6^ Similarly, tip-enhanced catalytic efficiency is influenced by the shape and species of the tip.^7,8^

Off-axis electron holography^9^ in the transmission electron microscope (TEM) is a powerful technique that can be used to map projected and three-dimensional (3D) electrostatic potentials,^10−18^ electric fields,^19−23^ and charge density distributions^24−28^ with nanometer to atomic spatial resolution. 3D electrostatic potentials have been measured from tilt series of electron holographic phase images to study dopant potentials in semiconductors,^11,12,14,15^ and the morphologies of nanoscale materials^29−31^ and 3D electric fields around specimens have been inferred from phase images based on the assumption of rotational symmetry^23,32^ or have made use of simple analytical models.^22^ Measurements of 3D charge density distributions in materials have also been proposed.^33,34^ However, until now only empirical models for charge density distributions within samples have been fitted by comparing simulated projected potentials with experimental phase images, for example, by making use of a line-charge model and the assumption of cylindrical symmetry for reconstruction of the 3D electric field around a metallic atom probe needle.^22^

In general, the use of back-projection-based tomographic reconstruction to study 3D electrostatic potentials and electric fields is complicated by the fact that they are continuous functions that may vary significantly in magnitude within and outside both the specimen and the field of view (FOV). Reconstructions are then affected more significantly by artifacts than when using electron tomography to recover variations in morphology and composition in materials, especially if full 360° rotation cannot be achieved when recording an experimental tomographic tilt series of off-axis electron holograms. If electrostatic fringing fields are present outside the specimen, then direct tomographic reconstructions of potentials and fields are also affected by the so-called perturbed reference wave (PRW) effect in off-axis electron holography.^23,35^

In this paper, we show that these problems can be circumvented by not determining the 3D potential or field directly, but by combining electron holographic tomography with model-based iterative reconstruction (MBIR)^28,36^ to first determine the 3D charge density in the specimen, i.e., the source of the potential and field. This approach allows *a priori* information, such as the shape of the object, the PRW effect, and the influence of charges that are located outside the FOV to be taken into account.^28^ It is also more robust to noise and artifacts that originate from the use of a limited tilt range and can provide a quantitative number for the spatial resolution of the reconstructed charge density.^34,36^ The reconstructed 3D charge distribution in the specimen can then be used to infer the 3D electrostatic potential and electric field both inside and around the specimen without the artifacts that would be present by reconstructing them from recorded phase images directly.

We illustrate the method through the experimental measurement of the 3D charge density in an electrically biased C fiber needle that has a long-range electric field outside it with a spatial resolution of approximately 5 nm. As a needle-shaped sample geometry is similar to that present in some nanoelectronic devices, such as semiconducting nanowires, nanoscale p*–*n junctions, and field emitters,^11,13,15,20,37^ we believe that our results will also be useful in these research fields, in particular to understand the relationship between local electrical properties and the presence of defects, dopants, interfaces, and surfaces.

A C fiber needle was prepared using a standard focused ion beam (FIB) milling procedure in an FEI Helios Nanolab 460F1 workstation, as described elsewhere in detail.^38^ An electrical bias was applied between the needle and a micrometer-sized Au counter electrode in the TEM using a Nanofactory scanning tunneling microscopy TEM specimen holder. Figure 1a shows a low-magnification bright-field TEM image of the experimental setup. The length of the C fiber needle is approximately 2.5 μm. The distance between the needle and the Au counter electrode is approximately 4.5 μm. The diameter of the apex of the needle is approximately 60 nm. A higher magnification bright-field TEM image is shown in Figure 1b.

**Figure 1:**
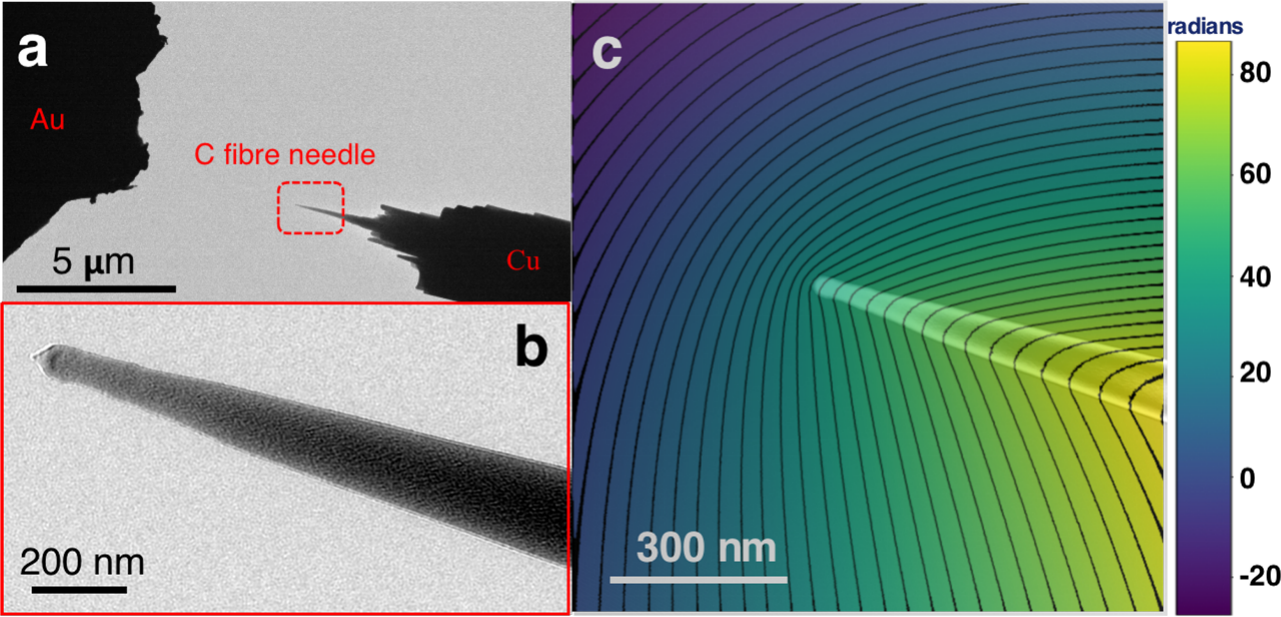
(a) Low-magnification bright-field TEM image showing the experimental setup for electrical biasing of a C fiber needle in the TEM. (b) Higher magnification image of the end of the needle. (c) Electron holographic phase difference image with +40 V applied between the needle and the counter electrode at a 0° sample tilt angle. The mean inner potential contribution to the phase has been removed by subtracting an aligned phase image recorded without a bias applied to the needle. Equiphase contour lines (black) are superimposed. The phase contour spacing is 2π radians. A TEM image of the C fiber needle is overlaid to guide the eye.

As off-axis electron holography is sensitive to contributions to the electrostatic potential from both the mean inner potential (MIP) of the specimen and the applied electrical bias, two tomographic tilt series of electron holographic phase images were recorded over a tilt range of −52° to +48° with a tilt increment of 4°. The first tilt series was recorded without an electrical bias applied to the needle. On the assumption that the needle does not undergo electron-beam-induced charging, these phase images are sensitive only to the projected MIP of the specimen. They can be used to subtract the MIP contribution to the phase from subsequent images recorded at each tilt angle as well as to reconstruct the 3D shape of the needle. The second tilt series was recorded with +40 V applied between the needle and the counter electrode. The MIP contribution to the phase was removed by aligning and subtracting corresponding phase images at each tilt angle with and without the electrical bias applied to the needle. Figure S2 contains further details about this procedure.

The spatial resolution of the reconstruction presented below is limited to approximately 5 nm here primarily by the spatial resolution of the TEM and the aperture size used for hologram reconstruction. It can be improved in future studies by choosing an operating mode of the TEM that has higher spatial resolution, by using a larger aperture size for hologram reconstruction and by using approaches such as double-resolution or phase-shifting holography.^39,40^

Figure 1c shows a resulting phase difference image for a 0° sample tilt angle with equiphase contour lines superimposed. The asymmetry in the contours between the two sides of the needle is a consequence of the PRW effect, i.e., the influence on the holographic reference wave of the long-range electric field associated with the voltage applied to the needle. Figure S3 shows similar phase contour images for selected other sample tilt angles. The first data set alone was used to reconstruct the 3D shape of the needle using the ASTRA toolbox^41^ (see Figure S4 for further details).

3D reconstruction of the charge density in the electrically biased needle was performed by applying the MBIR approach^28,36^ to the aligned tomographic data set of phase difference images (after removal of the MIP contribution to the phase at each tilt angle). The 3D shape of the needle (reconstructed from the first data set) was used to define the volume in which charges can be placed during reconstruction, on the assumption that no charges are present in the vacuum region around the needle. A buffer region of voxels was also defined immediately outside the 3D reconstruction volume. This buffer region was also allowed to contain “artificial” charges, which are used to represent contributions from the PRW and from charges that are located outside the FOV. These charges can be removed after reconstruction when analyzing the charges in the needle alone. By varying the positions and magnitudes of the charges in the reconstruction volume and using them to obtain predicted phase images, the algorithm attempts to minimize the residual with the experimental phase difference images at each sample tilt angle. At the same time, it uses a Euclidean norm for regularization. This choice favors a solution that minimizes the norm of the charge density. In a conductor, charges are expected to be located on the specimen surface. Here, the total of the norm of the charge density is a measure of the total electrostatic potential energy.^42^ Therefore, the algorithm converges to a solution that corresponds to a minimum in electrostatic energy. To an extent, this approach guarantees the physical uniqueness of the solution. A compromise between the two criteria is implemented by using a standard L-curve analysis. An optimized parameter for the regularization is chosen in order to balance the residual error between the experimental and reconstructed phase measurements and the minimum of the Euclidean norm of the reconstructed charges. Further details are available in Figure S5 and elsewhere.^28,36^

Figure 2a shows a visualization of the resulting reconstructed 3D charge density in the C fiber needle. As expected, the charge density is greatest at the apex of the needle, which has the highest curvature and is closest to the Au counter electrode. The maximum charge density is 2.94 × 10^18^ cm^–3^. The charge is distributed asymmetrically around the needle, which is not perfectly circular in cross section. Such an effect can also originate from the reconstruction algorithm if the mask that defines the sample surface is not chosen correctly or if the boundary buffer voxel approach that is used to represent charges outside the FOV and the PRW effect does not work effectively. We therefore verified the result by evaluating the influence on the reconstructed charge density of changing the size of the 3D mask manually as well as changing the thickness of the buffer region from 8 to 16, 32, and 64 voxels.

**Figure 2:**
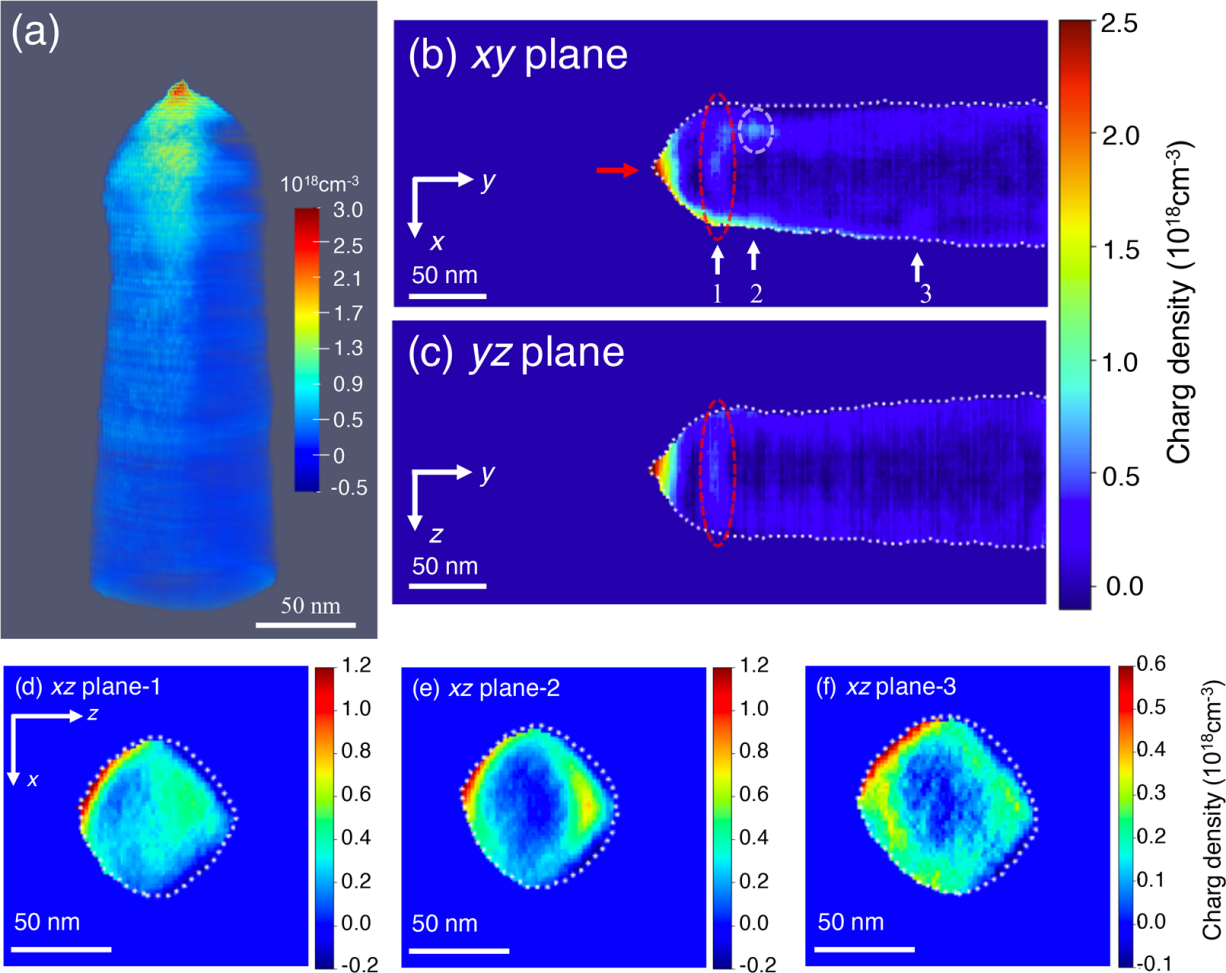
Visualization of 3D charge density in a C fiber needle reconstructed using electron holographic tomography and the MBIR approach. The needle is biased at +40 V with a distance of 4.5 μm from a Au counter electrode. (a) Side view of the 3D charge density. (b, c) 2D slices of charge density in the (b) *xy* and (c) *yz* planes, extracted from the 3D reconstructed charge density shown in (a). (d–f) Three representative slices of charge density in the *xz* plane extracted from the 3D reconstructed charge density shown in (a). The positions of the planes are marked by white arrows and numbers in (b). The white dashed lines mark the outline of the needle. See text for details.

Selected slices extracted from the reconstructed 3D charge density distribution are shown in Figures 2b–f. The *xy* plane (Figure 2b) reveals an asymmetry in the charge density (white arrow “1”), which is absent in the *yz* plane (Figure 2c) and may be associated with local differences in the shape of the needle. The fact that the majority of the charge is located close to the surface of the needle is visible in the *yz* plane (Figure 2c). On the one hand, this observation is consistent with the prediction (based on classical electrostatics) that charges reside on the outer surface of a conductor.^42^ On the other hand, it is surprising that the charge penetrates several tens of nanometers into the surface, perhaps because the needle has a disordered or poorly conducting surface layer. As the spatial resolution of our method is currently about 5 nm, it does not provide unambiguous information about field penetration, band bending, or screening depth.^43^ This information should be accessible in the future when the spatial resolution of the technique is improved.

A region with a local increase in charge density is marked by red dashed ellipses in Figures 2b,c. It may be associated with a local difference in damage to the needle during sample preparation (e.g., Ga^+^ bombardment). Figures 2d–f show three representative *xz* planes (labeled in Figure 2b), in which the charge can be seen to be located primarily at the surface of the needle. Plane “1” is thought to lie at the interface between a more insulating apex region and the more conductive shank of the needle. Plane “2” intersects a local maximum in charge density (marked by a white dashed ellipse in Figure 2b). Plane “3” corresponds to a plane that is at a greater distance from the apex. In planes “2” and “3”, the charge is localized at the outer surface of the needle.

Corresponding charge density profiles determined from the 3D reconstruction are shown in Figure 3. Figure 3a shows a line profile of the charge density in the *xy* plane along the axis of the needle (marked by a red arrow in Figure 2b). In addition to a maximum at the apex, there is a local maximum with a charge density of approximately 0.5 × 10^18^ cm^–3^. The charge density along the axis of the rest of needle is close to zero. For the three chosen *xz* planes, line profiles of the measured charge density are plotted in the *z* and *x* directions in Figures 3b and 3c, respectively. In the *z* direction (Figure 3b), the green and blue profiles from planes “2” and “3” are almost symmetrical with respect to the needle axis. The red profile from plane “1” is higher than those from planes “2” and “3” on the axis of the needle. In the *x* direction (Figure 3c), the red profile from plane “1” is again higher on the axis of the needle than the other two profiles. It should be noted that negative values in the reconstructed charge distribution fall within the errors that are present due to noise from the experimental data and the reconstruction.

**Figure 3:**
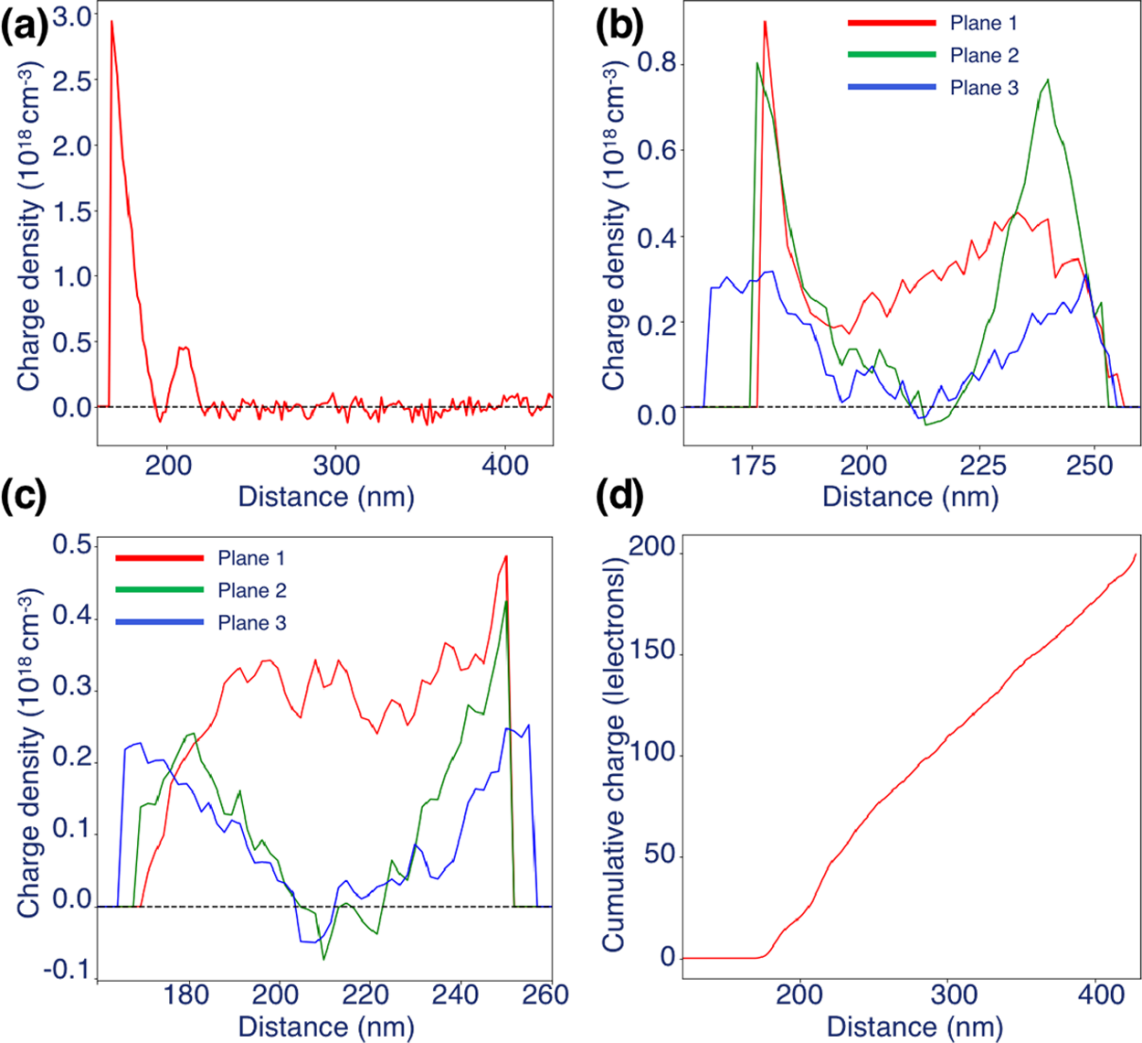
Line profiles of charge density extracted from the 3D charge density shown in Figure 2a along the axis of the needle (*y*) in the central *xy* plane (marked by a red arrow in Figure 2b) and (b, c) along *z* and *x* in three different *xz* planes (1: red; 2: green; 3: blue) marked in Figure 2b. (d) Cumulative charge profile along the length of the needle.

The cumulative charge integrated along the length of the needle, which is shown in Figure 3d, follows an almost linear trend, suggesting that the line charge density along the needle is almost constant, as expected from analytical solutions.^21,35^ The slope of the cumulative charge profile is slightly greater close to the apex of the needle than in the shank, indicating a greater line charge density in this region, perhaps because of its more insulating character or because of a local deviation from an ellipsoidal geometry.^44^

The 3D electrostatic potential and electric field can be calculated from the 3D reconstructed charge density by removing the artificial charges in the buffer voxel region and assuming that image charges in the counter electrode can be defined to have a norm vector with respect to the apex of the needle of (0,–4.5, 0) μm, as determined from the experimental setup (Figure 1b). It should be noted that charges outside the FOV also contribute to the electrostatic potential and electric field inside the FOV. However, as the electric field decays in a quadratic manner with distance and the charge density in the needle is negligible far from the apex (see Figure 3d), it is reasonable to assume that charges outside the FOV have a negligible contribution in this setup. This consideration is not in conflict with simulations performed by the atom probe community (e.g.,^45,46^), as we are focusing here only on the local electric field within the FOV, whereas additional boundary conditions in an atom probe apparatus (further from the tip of the needle) affect ion trajectories to the detector.

Figure 4a show a 3D visualization of the magnitude of the electric field, while Figures 4b,c show slices of the magnitude of the electric field in the central *xy* and *yz* planes. The electric field is almost symmetrical about the needle axis. Residual slight asymmetry results from the asymmetry in the reconstructed charge density (as seen in the *xy* plane in Figure 4b). Figures 4b,c show that the electric field is strongest close to the apex and that it decays rapidly into the vacuum region with increasing distance from it. The maximum electric field strength measured in this study is approximately 0.25 GV/m. On the basis of a single projection model^6^ used by the atom probe community, the geometric field factor here is predicted to be approximately 5.33, which falls within theoretical values that typically range between 2 and 14.^46^ It should be noted that this factor is highly dependent on the radius and shape of the tip and on the distance to the counter electrode. Inside the needle (Figures 4b,c), the magnitude of the electric field is strongest in the region close to the surface, where it is thought to be less conducting.

**Figure 4:**
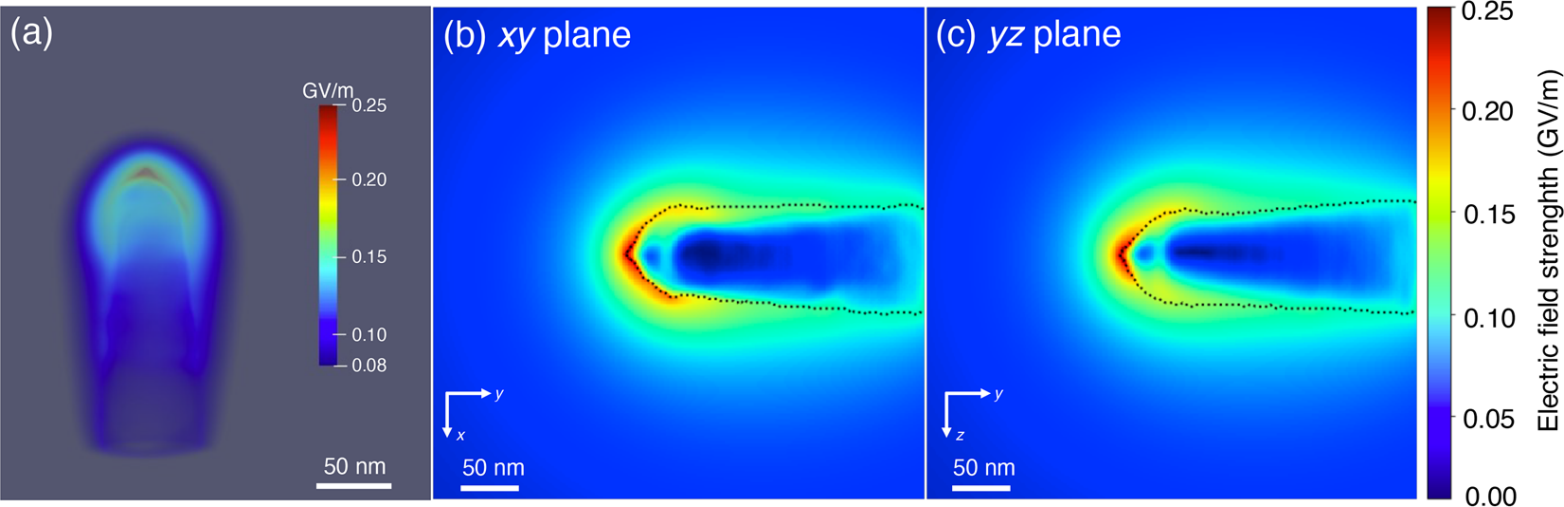
3D electric field determined from the reconstructed 3D charge density shown in Figure 2 for the electrically biased C fiber needle. (a) Magnitude of the electric field plotted on a logarithmic scale. (b, c) Magnitude of the electric field in the central *xy* and *yz* planes, respectively. The black dashed line shows the outline of the needle.

A combination of a streamline plot of the electric field and the electrostatic potential in the central *xy* plane is shown in Figure 5. The fact that the field lines are normal to the surface of the needle close to its apex suggests that charges outside the FOV do not influence the reconstruction in this region and that the electric field around the apex can be calculated reliably using the MBIR approach. In contrast, the field lines on the right of the image (close to the shank of the needle) are inclined with respect to the surface of the needle as a result of the influence of missing contributions to the electric field from charges outside the FOV.

**Figure 5:**
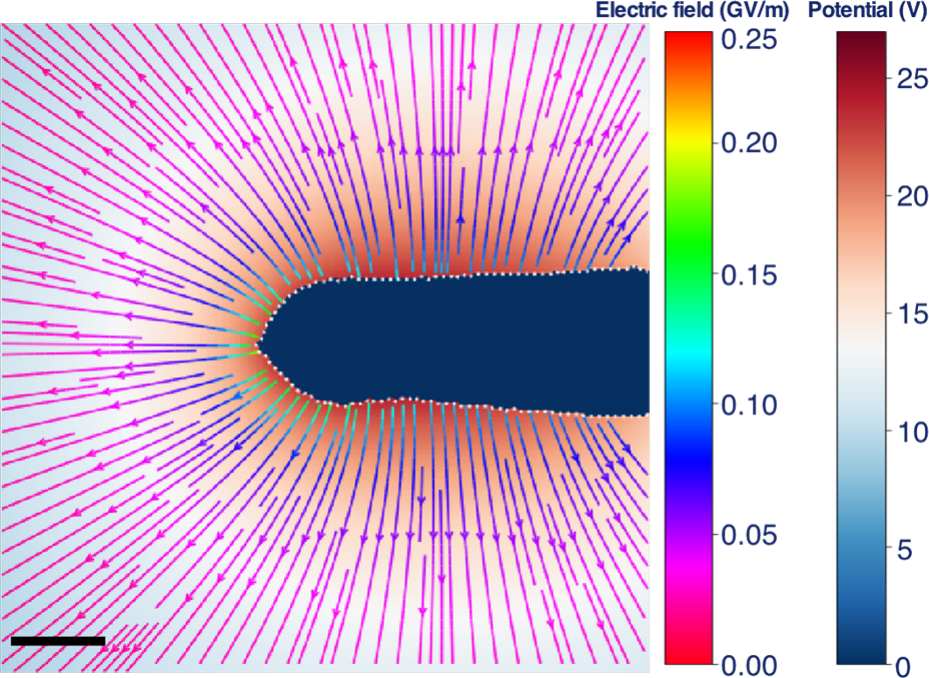
Combination of a streamline plot of the electric field and a plot of the electrostatic potential in the central *xy* plane of the reconstructed volume of the C fiber needle. The interior of the needle is marked in blue. The scale bar is 50 nm.

In summary, the 3D charge density, electrostatic potential, and electric field of an electrically biased C fiber needle have been determined experimentally using electron holographic tomography combined with model-based iterative reconstruction of the charge density. Tomographic tilt series of holographic phase images with the needle biased at +40 V with respect to a Au counter electrode were used as input for the algorithm after subtracting the MIP contribution to the phase from each image in the tilt series. The reconstructed charge density is greatest at the apex of the needle and localized primarily close to its surface. The cumulative charge displays a close-to-linear behavior, which is consistent with a line charge density that is almost constant along the needle. Asymmetry in the reconstructed charge density results from changes in the local geometry of the needle or damage from FIB sample preparation. The 3D electric field and electrostatic potential can be calculated from the reconstructed 3D charge density based on an assumption for the positions of image charges in the Au counter electrode. The inferred electric field and electrostatic potential are both almost symmetrical about the needle axis. The strength of the electric field is greatest close to the apex of the needle and has a maximum value of 0.25 GV/m at an applied bias voltage of +40 V with a distance of 4.5 μm to the Au counter electrode. The results suggest that this approach can be used for 3D characterization of charge, electrostatic potential, and electric field from limited data sets recorded from a wide range of nanostructures and may provide guidance for designs and improvements of nanoscale electronic devices.

## Methods

### C Fiber

A high-strength C fiber (T1000) was obtained from Toray Company (www.toray.jp). The fiber comprised primarily C (≥99%), with a small amount of N, in the form of a highly textured structure along the fiber axis, with turbostratic graphene as the basic structural unit.^47^ This choice of material reduces the influence of changing diffraction conditions during tomographic tilt series acquisition.

### Off-Axis Electron Holography

An FEI Titan G^2^ 60*-*300 TEM equipped with an ultrabright-field emission electron gun (X-FEG) and two electrostatic biprisms was used for off-axis electron holography experiments. The operating voltage was set to 300 kV. In order to obtain a large field of view, experiments were performed in Lorentz mode with the conventional microscope objective lens switched off. A scanning tunneling microscopy TEM specimen holder (Nanofactory Instruments) was used for electrical biasing experiments. Off-axis electron holograms were recorded using an exposure time of 6 s on a 4k × 4k direct electron counting Gatan K2 IS camera. A representative hologram is shown in Figure S1. The holographic interference fringe spacing was approximately 1.7 nm, and the interference width was approximately 1.8 μm. Thirty holograms were recorded at each tilt angle to increase the signal-to-noise ratio. Reference holograms were recorded from a region of vacuum with the specimen removed from the field of view. Real-space phase and amplitude images were reconstructed from recorded electron holograms with a standard fast Fourier transform algorithm using Holoworks software (Holowerk LLC).

## Supporting Information Available

Representative off-axis electron hologram, reconstructed phase images, phase contour maps, reconstructed 3D shape of the needle and parameters used for model-based iterative reconstruction (PDF)
