# Supplementary material for: Nanoscale Three-Dimensional Charge Density and Electric Field Mapping by Electron Holographic Tomography
Source: Nano Lett. 2023 Jan 23;23(3):843–9. doi: 10.1021/acs.nanolett.2c03879 (PMC9912371; doi:10.1021/acs.nanolett.2c03879)
Supplement: Supplementary file 1 — nl2c03879_si_001.pdf [file nl2c03879_si_001.pdf]

Supporting Information for "Nanoscale three-dimensional charge density and electric field mapping by electron holographic tomography"

Fengshan Zheng,[*,†,‡] Vadim Migunov,[†,¶] Jan Caron,[†] Hongchu Du,[†,¶] Giulio Pozzi,[†,§] and Rafal E. Dunin-Borkowski[†]

†*Ernst Ruska-Centre for Microscopy and Spectroscopy with Electrons and Peter Grünberg Institute, Forschungszentrum Jülich, 52425 Jülich, Germany*

‡*Spin-X Institute, Electron Microscopy Center, School of Physics and Optoelectronics, State Key Laboratory of Luminescent Materials and Devices, Guangdong-Hong Kong-Macao Joint Laboratory of Optoelectronic and Magnetic Functional Materials, South China University of Technology, Guangzhou 511442, China*

¶*Central Facility for Electron Microscopy (GFE), RWTH Aachen University, Ahornstrasse 55, 52074 Aachen, Germany*

§*Department FIM, University of Modena and Reggio Emilia, via G. Campi 213/a, 41125 Modena, Italy*

E-mail: f.zheng@fz-juelich.de

Representative off-axis electron hologram

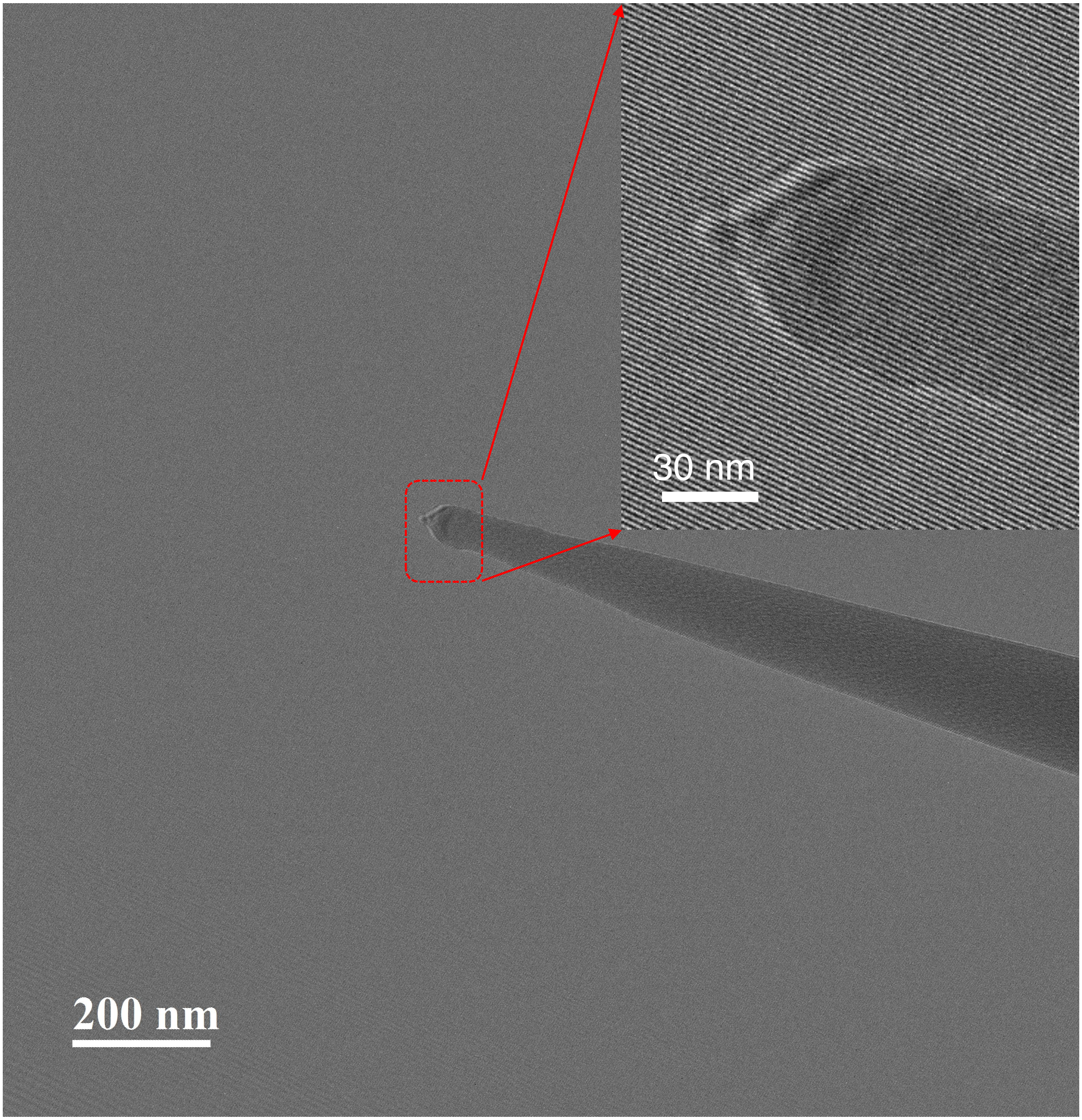

Figure 1: Representative off-axis electron hologram with the needle electrically biased at +40 V. The inset shows magnified holographic interference fringes around the apex region.

Representative phase images

Figure 2a shows the mean inner potential (MIP) contribution to the phase of the needle with no applied bias voltage and a corresponding phase contour map. The flatness of the vacuum region indicates that electron-beam-induced charging of the needle is negligible. Figure 2b shows a phase image recorded with the needle biased at +40 V and a corresponding phase contour map. The asymmetry of the phase contours results from the PRW effect. Figure 2c shows the difference between the phase images (shown in Figs 2a-b) after alignment and a corresponding phase difference contour map. The difference image represents the contribution to the phase from the electrical bias alone.

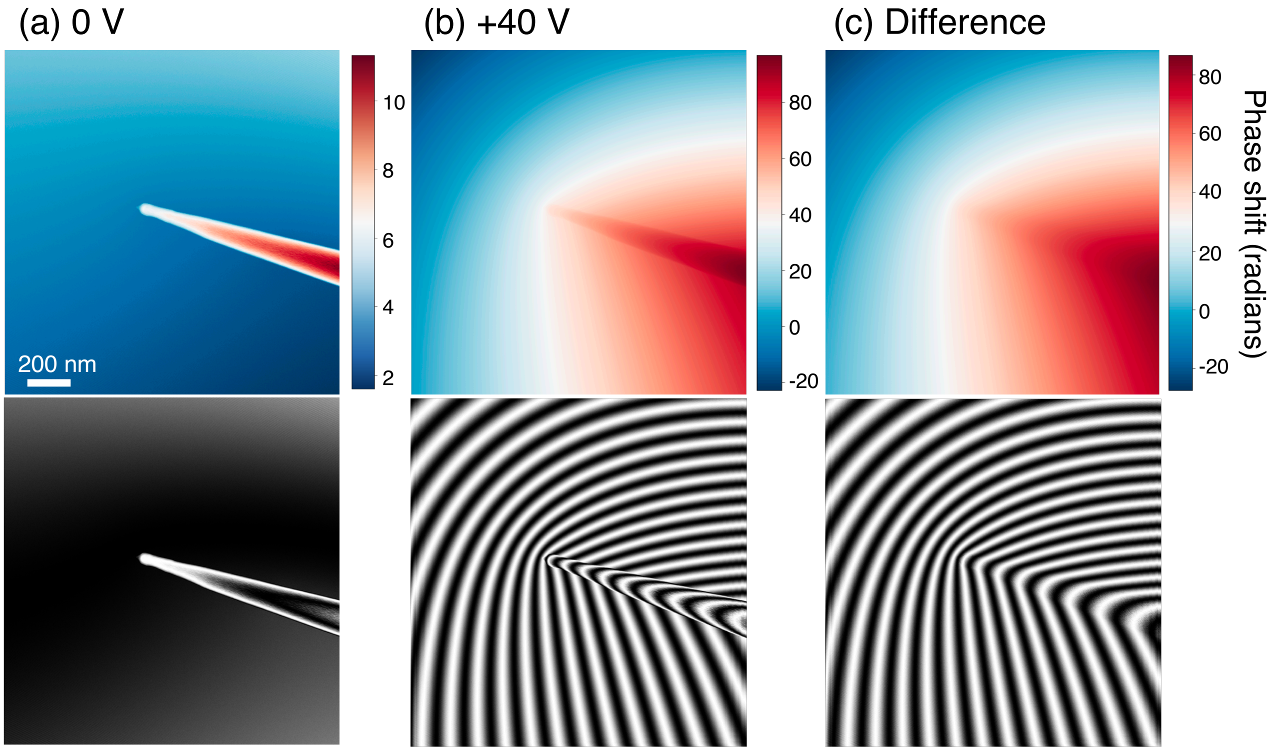

Figure 2: Phase images (top) and corresponding phase contour maps (bottom) recorded with the needle biased at 0 and +40 V and their difference. (a) MIP contribution to the phase (0 V). (b) Phase including contributions from the MIP and the electrical bias at +40 V. (c) Difference between (a) and (b) after alignment. The sample tilt angle was 0°. The phase contour spacing is 2π radians.

Selected phase difference contour maps

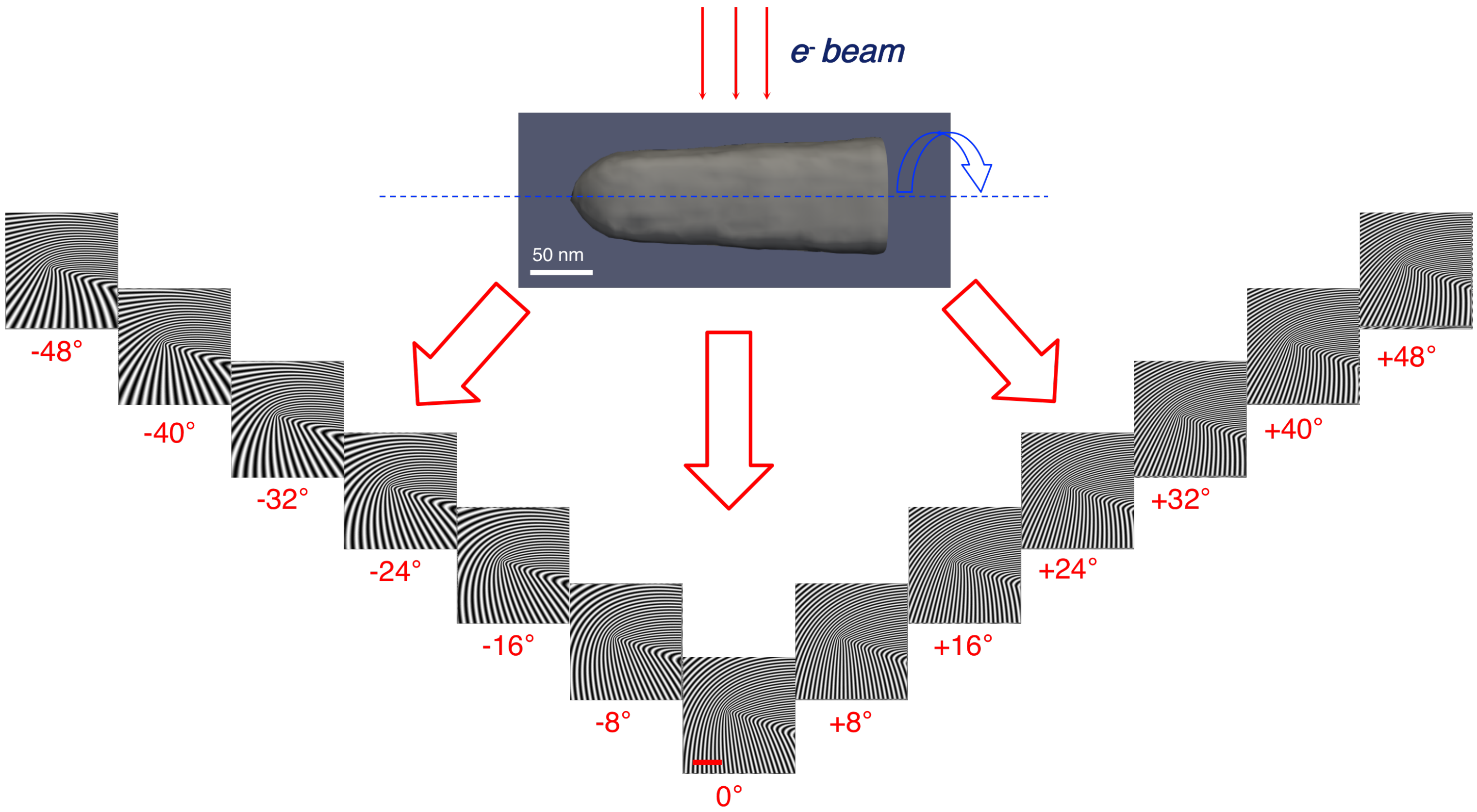

Figure 3: Selected phase difference contour maps generated from a tomographic tilt series of off-axis electron holograms with the needle electrically biased at +40 V. The sample tilt angle is indicated next to each image. The phase contour spacing is π radians. The MIP contribution to the phase was removed before calculating each map. In the full tilt series, the tilt increment is 4° and the tilt angle range is from $-52°$ to $+48°$. The scale bar (shown in the 0°) image) is 400 nm.

3D shape of the needle

The successful application of the MBIR approach to reconstruct the 3D charge density requires the use of a 3D mask to define where the charge can be located in the reconstruction.[1] Since the charge can only be located inside the needle, its outer surface can serve as a 3D mask. Such a mask can be obtained from a tomographic reconstruction of the MIP contribution to the phase. Here, such images were used to generate a 3D mask by using the ASTRA toolbox.[2] A discrete algebraic reconstruction tomography (DART) algorithm was used to avoid artefacts originating from the missing wedge.[3] Figure 4 shows the reconstructed 3D shape of the C fibre needle. The reconstruction shows that the needle-shaped geometry (side view in Fig. 4a) has a rounded rectangular cross-section (top view in Fig. 4b). Two central slices of the xy and yz planes, which are shown in Figs 4c-d, respectively, indicate slight asymmetry in the x and z directions of the cross-section of the needle.

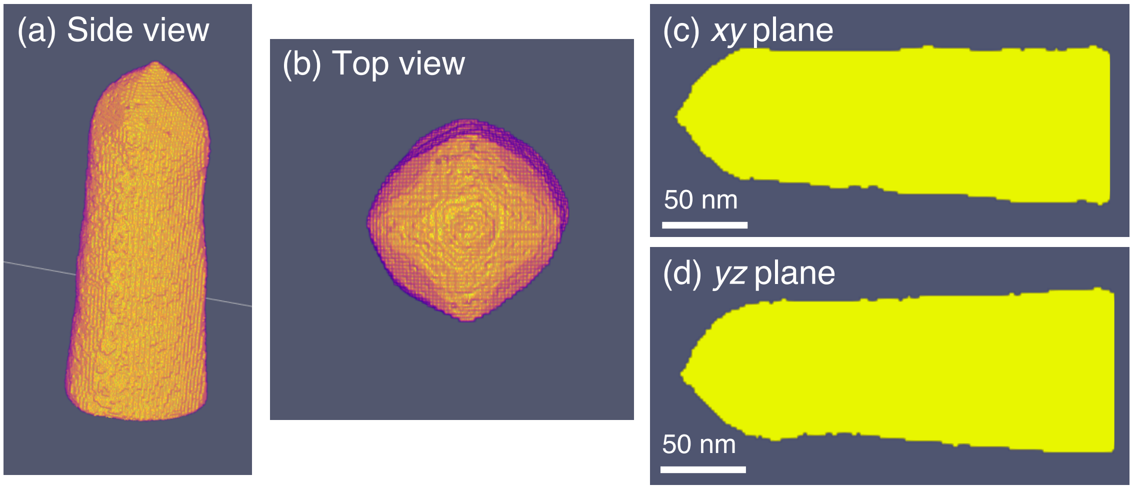

Figure 4: 3D shape of the C fibre needle reconstructed from a tomographic tilt series of the MIP contribution to the phase, showing (a) a side view and (b) a top view. (c, d) Central slices of the xy and yz planes. The needle is marked in yellow. The reconstructed surface was used as a 3D mask for model-based iterative reconstruction of charge inside the needle-shaped specimen. See text for details.

Input parameters for model-based iterative reconstruction

The sampling density of the holograms was approximately 1.7 nm and the voxel size in the reconstruction was also chosen to be 1.7 nm. However, the true spatial resolution of each reconstructed phase image was limited (by the size of the sideband used for reconstruction) to approximately 5 nm. The 3D shape of the needle was used as a mask to define where charges can be located. A tomographic tilt series of phase difference images (with the MIP contribution to the phase removed) was used as input for reconstruction. The position of the surface normal vector of the counter-electrode was measured experimentally to be approximately (-4.5, 0, 0) μm from the apex of the needle and the counter-electrode. An 8-pixel-wide buffer region was used to take into account contributions from charges outside the FOV and the PRW effect. The optimal regularisation parameter was set to 1000 based on an L-curve analysis (Fig. 5).

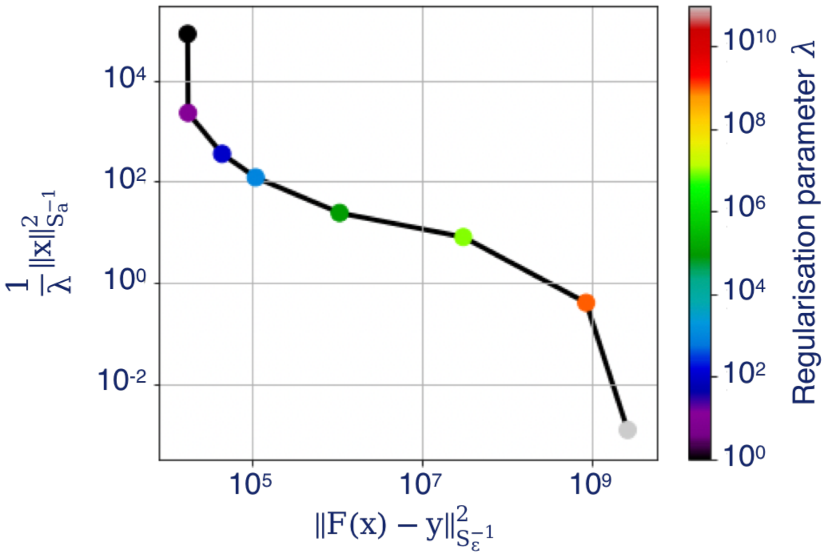

Figure 5: L-curve analysis of the 3D reconstruction from a tomographic tilt series of phase difference images of the C fibre needle. The optimal regularisation parameter λ was chosen to be 1000.

References

(1) Zheng, F.; Caron, J.; Migunov, V.; Beleggia, M.; Pozzi, G.; Dunin-Borkowski, R. E. Measurement of charge density in nanoscale materials using off-axis electron holography. *Journal of Electron Spectroscopy and Related Phenomena* **2020**, *241*, 146881.

(2) van Aarle, W.; Palenstijn, W. J.; De Beenhouwer, J.; Altantzis, T.; Bals, S.; Batenburg, K. J.; Sijbers, J. The ASTRA Toolbox: A platform for advanced algorithm development in electron tomography. *Ultramicroscopy* **2015**, *157*, 35–47.

(3) Saghi, Z.; Divitini, G.; Winter, B.; Leary, R.; Spiecker, E.; Ducati, C.; Midgley, P. A. Compressed sensing electron tomography of needle-shaped biological specimens–Potential for improved reconstruction fidelity with reduced dose. *Ultramicroscopy* **2016**, *160*, 230–238.
